# Supplementary material for: Epidemiology of Plasmodium malariae and Plasmodium ovale spp. in Kinshasa Province, Democratic Republic of Congo
Source: Nat Commun. 2023 Oct 19;14:6618. doi: 10.1038/s41467-023-42190-w (PMC10587068; doi:10.1038/s41467-023-42190-w)
Supplement: Supplementary file 1 — Supplementary Information [file 41467_2023_42190_MOESM1_ESM.pdf]

SUPPLEMENT TO:

Epidemiology of *Plasmodium malariae* and *Plasmodium ovale* spp. in Kinshasa Province, Democratic Republic of Congo

Authors: Rachel Sendor[1,*], Kristin Banek[2], Melchior Mwandagalirwa Kashamuka[3], Nono Mvuama[3], Joseph A. Bala[3], Marthe Nkalani[3], Georges Kihuma[3], Joseph Atibu[3], Kyaw L. Thwai[2], W. Matthew Svec[4], Varun Goel[5,6], Tommy Nseka[3], Jessica T. Lin[2,7], Jeffrey A. Bailey[8], Michael Emch[1,5,6], Margaret Carrel[9], Jonathan J. Juliano[1,2,7], Antoinette Tshefu[3,¶], Jonathan B. Parr[2,7,¶]

Affiliations:

[1] Department of Epidemiology, Gillings School of Global Public Health, University of North Carolina at Chapel Hill, Chapel Hill, North Carolina, United States of America

[2] Institute for Global Health and Infectious Diseases, University of North Carolina at Chapel Hill, Chapel Hill, North Carolina, United States of America

[3] Ecole de Santé Publique, Faculté de Médecine, University of Kinshasa, Kinshasa, Democratic Republic of the Congo

[4] University of North Carolina at Chapel Hill, Chapel Hill, North Carolina, United States of America

[5] Department of Geography, University of North Carolina at Chapel Hill, Chapel Hill, North Carolina, United States of America

[6] Carolina Population Center, University of North Carolina at Chapel Hill, Chapel Hill, North Carolina, United States of America

[7] Division of Infectious Diseases, School of Medicine, University of North Carolina at Chapel Hill, Chapel Hill, North Carolina, United States of America

[8] Department of Pathology and Laboratory Medicine and Center for Computational Molecular Biology, Brown University, Providence, Rhode Island, United States of America

[9] Department of Geographical and Sustainability Sciences, University of Iowa, Iowa City, Iowa, United States of America

* Corresponding authors: E-mail: rachel.sendor@unc.edu (RS); jonathan_parr@med.unc.edu (JBP)

¶ These authors jointly supervised this work.

Supplementary Table 1. Comparison of participant characteristics between survey population and clinic subpopulation.

Participant Baseline Characteristics *no. (%)*	Population Type[1]				
	Survey-based Population	Also in Clinic-based Subpopulation?			
	n=1,565 participants	Yes n=1,050 (67.1%)	No n=515 (32.9%)	p-value[3]	SMD
Age at visit (years)					
<5	302 (19.3)	229 (21.8)	73 (14.2)	<0.001	0.276
5-14	500 (31.9)	354 (33.7)	146 (28.3)		
15+	763 (48.8)	467 (44.5)	296 (57.5)		
Missing	0	0	0		
Sex					
Female	863 (55.1)	587 (55.9)	276 (53.6)	0.388	0.046
Male	702 (44.9)	463 (44.1)	239 (46.4)		
Missing	0	0	0		
Site					
Voix de Peuple	385 (24.6)	230 (21.9)	155 (30.1)	<0.001	0.351
Bu	209 (13.4)	171 (16.3)	38 (7.4)		
Impuru	211 (13.5)	136 (13.0)	75 (14.6)		
Pema	243 (15.5)	180 (17.1)	63 (12.2)		
Kimpoko	167 (10.7)	111 (10.6)	56 (10.9)		
Ngamanzo	258 (16.5)	167 (15.9)	91 (17.7)		
Iye	92 (5.9)	55 (5.2)	37 (7.2)		
Missing	0	0	0		
Rurality (by health area)					
Urban	385 (24.6)	230 (21.9)	155 (30.1)	<0.001	0.262
Peri-urban	517 (33.0)	333 (31.7)	184 (35.7)		
Rural	663 (42.4)	487 (46.4)	176 (34.2)		
Missing	0	0	0		
Wealth quintile					
Poorest	318 (20.3)	226 (21.5)	92 (17.9)	0.011	0.194
Poorer	314 (20.1)	209 (19.9)	105 (20.4)		
Average	311 (19.9)	223 (21.2)	88 (17.1)		
Wealthier	304 (19.4)	202 (19.2)	102 (19.8)		
Wealthiest	318 (20.3)	190 (18.1)	128 (24.9)		
Missing	0	0	0		
Fever at Baseline[2]					
Yes	382 (24.5)	272 (26.0)	110 (21.5)	0.052	0.106
No	1176 (75.5)	774 (74.0)	402 (78.5)		
Missing	7	4	3		
RDT+ at Baseline					
Yes	429 (27.4)	285 (27.2)	144 (28.0)	0.77	0.019
No	1134 (72.6)	764 (72.8)	370 (72.0)		
Missing	2	1	1		
Seasonality of visit					
Rainy	1180 (75.4)	820 (78.1)	360 (69.9)	0.001	0.188

Participant Baseline Characteristics *no. (%)*	Survey-based Population n=1,565 participants	Also in Clinic-based Subpopulation? Yes n=1,050 (67.1%)	No n=515 (32.9%)	p-value[3]	SMD
Dry	385 (24.6)	230 (21.9)	155 (30.1)		
Missing	0	0	0		
Slept under bednet at Baseline					
Yes	705 (45.0)	480 (45.7)	225 (43.7)	0.482	0.041
No	860 (55.0)	570 (54.3)	290 (56.3)		
Missing	0	0	0		
Antimalarials taken within prior 6 months					
Yes	394 (25.4)	277 (26.6)	117 (22.8)	0.119	0.088
No	1160 (74.6)	764 (73.4)	396 (77.2)		
Missing	11	9	2		
P. falciparum PCR +					
Yes	484 (30.9)	314 (29.9)	170 (33.0)	0.222	0.067
Mixed-species	*35 (7.2)*	*25 (8.0)*	*10 (5.9)*	*0.51*	*0.082*
Single-species	*449 (92.8)*	*289 (92.0)*	*160 (94.1)*		
No	1081 (69.1)	736 (70.1)	345 (67.0)		
Missing	0	0	0		
P. malariae PCR +					
Yes	47 (3.0)	33 (3.1)	14 (2.7)	0.753	0.025
Mixed-species	*31 (66.0)*	*22 (66.7)*	*9 (64.3)*	*1*	*0.05*
Single-species	*16 (34.0)*	*11 (33.3)*	*5 (35.7)*		
No	1518 (97.0)	1017 (96.9)	501 (97.3)		
Missing	0	0	0		
P. ovale spp. PCR +					
Yes	6 (0.4)	5 (0.5)	1 (0.2)	0.67	0.049
Mixed-species	*4 (66.7)*	*3 (60.0)*	*1 (100.0)*	*1*	*1.155*
Single-species	*2 (33.3)*	*2 (40.0)*	*0 (0.0)*		
No	1559 (99.6)	1045 (99.5)	514 (99.8)		
Missing	0	0	0		

(Column group header: Population Type[1])

[1] The survey-based population comprises all participants in the study, as all participants completed the Baseline household survey. The clinic-based sub-population comprises a subset of the survey-based population who had at least 1 symptomatic clinic visit during the study period.
[2] Fever at Baseline survey was self-reported as "fever in the prior week"
[3] p-values compare baseline characteristics between those in the survey-based population who were vs. were not also included in the clinic-based symptomatic population. p-values were calculated using two-sided chi-square testing, or fishers' exact testing in the case of small frequency counts, and are not adjusted for multiple comparisons.

Supplemental Table 2. Baseline participant characteristics by species for the symptomatic clinic subpopulation[1]

Baseline Participant Characteristics	Clinic-based Pop.	Baseline Malaria Infection by Species					
		P. malariae		*P. ovale* spp.		*P. falciparum*	
		PCR Pos.	PCR Neg.	PCR Pos.	PCR Neg.	PCR Pos.	PCR Neg.
no. (%)	N=1,050	n=33	n=1,017	n=5	n=1,045	n=314	n=736
**Age (years)**							
<5	229 (21.8)	4 (12.1)	225 (22.1)	1 (20.0)	228 (21.8)	49 (15.6)	180 (24.5)
5-14	354 (33.7)	23 (69.7)	331 (32.5)	2 (40.0)	352 (33.7)	161 (51.3)	193 (26.2)
15+	467 (44.5)	6 (18.2)	461 (45.3)	2 (40.0)	465 (44.5)	104 (33.1)	363 (49.3)
**Sex**							
Female	587 (55.9)	19 (57.6)	568 (55.9)	5 (100.0)	582 (55.7)	165 (52.5)	422 (57.3)
Male	463 (44.1)	14 (42.4)	449 (44.1)	0 (0.0)	463 (44.3)	149 (47.5)	314 (42.7)
**Urbanicity[2]**							
Rural	487 (46.4)	21 (63.6)	466 (45.8)	5 (100.0)	482 (46.1)	197 (62.7)	290 (39.4)
Peri-urban	333 (31.7)	10 (30.3)	323 (31.8)	0 (0.0)	333 (31.9)	110 (35.0)	223 (30.3)
Urban	230 (21.9)	2 (6.1)	228 (22.4)	0 (0.0)	230 (22.0)	7 (2.2)	223 (30.3)
**Fever (≤1 week)**							
Yes	272 (26.0)	9 (28.1)	263 (25.9)	4 (80.0)	268 (25.7)	110 (35.3)	162 (22.1)
**RDT+**							
Yes	285 (27.2)	20 (62.5)	265 (26.1)	2 (40.0)	283 (27.1)	235 (75.1)	50 (6.8)
**Bed Net Use (Prior Night)**							
Yes	480 (45.7)	9 (27.3)	471 (46.3)	2 (40.0)	478 (45.7)	127 (40.4)	353 (48.0)
**Symptoms in Prior 6 mon.**							
Yes	269 (25.7)	7 (21.9)	262 (25.8)	3 (60.0)	266 (25.5)	81 (26.0)	188 (25.6)
**Tx with Antimalarials in Prior 6 mon.**							
Yes	277 (26.6)	7 (21.9)	270 (26.8)	1 (20.0)	276 (26.6)	76 (24.4)	201 (27.6)
**Wealth Category**							
Poorest	226 (21.5)	5 (15.2)	221 (21.7)	1 (20.0)	225 (21.5)	93 (29.6)	133 (18.1)
Poorer	209 (19.9)	14 (42.4)	195 (19.2)	0 (0.0)	209 (20.0)	78 (24.8)	131 (17.8)
Average	223 (21.2)	6 (18.2)	217 (21.3)	2 (40.0)	221 (21.1)	69 (22.0)	154 (20.9)
Wealthier	202 (19.2)	6 (18.2)	196 (19.3)	2 (40.0)	200 (19.1)	69 (22.0)	133 (18.1)
Wealthiest	190 (18.1)	2 (6.1)	188 (18.5)	0 (0.0)	190 (18.2)	5 (1.6)	185 (25.1)

[1] The clinic population comprises a subset of the survey-based population who had a DBS at Baseline and had at least 1 symptomatic clinic visit during the study period. This table presents baseline characteristics only for the clinic subpopulation, overall, and broken down by whether the participant was PCR positive for each of the 3 *Plasmodium* species at the baseline visit.

[2] Bu health area is classified as rural, Kimpoko health area as peri-urban, and Voix du Peuple as urban.

Supplemental Table 3. Participant characteristics across follow-up visits (Survey population)

Participant characteristics at each visit *no. (%)*	Household Survey Visits (*Active surveillance*) Baseline n=1,565	Follow-up 1 n=1,447	Follow-up 2 n=1,367	Follow-up 3 n=1,303	p-value[3]
No. subjects	1,565	1,447	1,367	1,303	
Age at visit (years)					
<5	302 (19.3)	271 (18.7)	225 (16.5)	185 (14.2)	0.009
5-14	500 (31.9)	480 (33.2)	443 (32.4)	453 (34.8)	
15+	763 (48.8)	696 (48.1)	699 (51.1)	665 (51.0)	
Missing	*0*	*0*	*0*	*0*	
Sex					
Female	863 (55.1)	800 (55.3)	757 (55.4)	707 (54.3)	0.935
Male	702 (44.9)	647 (44.7)	610 (44.6)	596 (45.7)	
Missing	*0*	*0*	*0*	*0*	
Rurality (by health area)					
Urban	385 (24.6)	342 (23.6)	307 (22.5)	300 (23.0)	0.384
Peri-urban	517 (33.0)	487 (33.7)	457 (33.4)	402 (30.9)	
Rural	663 (42.4)	618 (42.7)	603 (44.1)	601 (46.1)	
Missing	*0*	*0*	*0*	*0*	
Wealth quintile					
Poorest	318 (20.3)	294 (20.3)	290 (21.2)	274 (21.0)	0.994
Poorer	314 (20.1)	288 (19.9)	277 (20.3)	264 (20.3)	
Average	311 (19.9)	301 (20.8)	292 (21.4)	271 (20.8)	
Wealthier	304 (19.4)	284 (19.6)	255 (18.7)	249 (19.1)	
Wealthiest	318 (20.3)	280 (19.4)	253 (18.5)	245 (18.8)	
Missing	*0*	*0*	*0*	*0*	
Fever[1]					
Yes	382 (24.5)	173 (12.0)	249 (18.2)	120 (9.2)	<0.001
No	1176 (75.5)	1273 (88.0)	1118 (81.8)	1183 (90.8)	
Missing	*7*	*1*	*0*	*0*	
RDT+					
Yes	429 (27.4)	396 (27.4)	511 (37.4)	307 (23.6)	<0.001
No	1134 (72.6)	1051 (72.6)	856 (62.6)	995 (76.4)	
Missing	*2*	*0*	*0*	*1*	
Slept under bednet the prior night					
Yes	705 (45.0)	810 (56.0)	732 (53.5)	589 (45.2)	<0.001
No	860 (55.0)	637 (44.0)	635 (46.5)	714 (54.8)	
Missing	*0*	*0*	*0*	*0*	
Seasonality of visit					
Rainy	1180 (75.4)	0 (0.0)	1365 (99.9)	19 (1.5)	<0.001
Dry	385 (24.6)	1447 (100.0)	2 (0.1)	1284 (98.5)	
Missing	*0*	*0*	*0*	*0*	
Antimalarials taken within prior 6 months[2]					
Yes	394 (25.4)	559 (38.9)	625 (46.1)	569 (46.6)	<0.001
No	1160 (74.6)	877 (61.1)	732 (53.9)	652 (53.4)	
Missing	*11*	*11*	*10*	*82*	
P. falciparum PCR +					
Yes	484 (30.9)	512 (35.5)	538 (39.6)	442 (34.1)	<0.001
Mixed-species	*35 (7.2)*	*42 (8.2)*	*64 (11.9)*	*47 (10.6)*	*0.043*

Participant characteristics at each visit *no. (%)*	Household Survey Visits (*Active surveillance)* Baseline n=1,565	Follow-up 1 n=1,447	Follow-up 2 n=1,367	Follow-up 3 n=1,303	p-value[3]
Single-species	*449 (92.8)*	*470 (91.8)*	*474 (88.1)*	*395 (89.4)*	
No	1081 (69.1)	930 (64.5)	820 (60.4)	853 (65.9)	
Missing	*0*	*5*	*9*	*8*	
P. malariae PCR +					
Yes	47 (3.0)	35 (2.4)	56 (4.1)	48 (3.7)	0.055
Mixed-species	*31 (66.0)*	*24 (68.6)*	*44 (78.6)*	*38 (79.2)*	*0.346*
Single-species	*16 (34.0)*	*11 (31.4)*	*12 (21.4)*	*10 (20.8)*	
No	1518 (97.0)	1408 (97.6)	1300 (95.9)	1247 (96.3)	
Missing	*0*	*4*	*11*	*8*	
P. ovale spp. PCR +					
Yes	6 (0.4)	27 (1.9)	27 (2.0)	18 (1.4)	<0.001
Mixed-species	*4 (66.7)*	*20 (74.1)*	*24 (88.9)*	*13 (72.2)*	*0.358*
Single-species	*2 (33.3)*	*7 (25.9)*	*3 (11.1)*	*5 (27.8)*	
No	1559 (99.6)	1416 (98.1)	1329 (98.0)	1277 (98.6)	
Missing	*0*	*4*	*11*	*8*	

[1] Fever was self-reported as "fever in the prior week"

[2] Self-reported use of antimalarials in prior 6 months.

[3] Categorical variables were statistically compared using two-sided chi-squared tests or fishers' exact testing in the case of small frequency counts; continuous variables were compared using a two-sided Kruskal-Wallis test of medians to account for non-normality. p-values were not adjusted for multiple comparisons. Missing data were excluded from statistical tests.

Supplemental Table 4. Participant characteristics across follow-up (Clinic Subpopulation)

Participant characteristics at visits *no. (%)*	Clinic Visits — Time from Baseline Visit[2]: **All visits within first 12 months** n=1297	**All visits between 12 to 24 months** n=1203	**All visits after 24 months** n=907	p-value[3]
No. subjects with visits	732	648	510	
Age at visit (years)				
<5	353 (27.2)	305 (25.4)	172 (19.0)	<0.001
5-14	481 (37.1)	434 (36.1)	386 (42.6)	
15+	463 (35.7)	462 (38.5)	348 (38.4)	
Missing	*0*	*2*	*1*	
Sex				
Female	741 (57.1)	726 (60.3)	514 (56.7)	0.153
Male	556 (42.9)	477 (39.7)	393 (43.3)	
Missing	*0*	*0*	*0*	
Site				
Voix de Peuple	182 (14.0)	213 (17.7)	109 (12.0)	<0.001
Bu	232 (17.9)	305 (25.4)	236 (26.0)	
Impuru	177 (13.6)	139 (11.6)	73 (8.0)	
Pema	261 (20.1)	203 (16.9)	162 (17.9)	
Kimpoko	217 (16.7)	114 (9.5)	104 (11.5)	
Ngamanzo	181 (14.0)	187 (15.5)	190 (20.9)	
Iye	47 (3.6)	42 (3.5)	33 (3.6)	
Missing	*0*	*0*	*0*	
Rurality (by health area)				
Urban	182 (14.0)	213 (17.7)	109 (12.0)	<0.001
Peri-urban	445 (34.3)	343 (28.5)	327 (36.1)	
Rural	670 (51.7)	647 (53.8)	471 (51.9)	
Missing	*0*	*0*	*0*	
Wealth quintile				
Poorest	288 (22.2)	235 (19.5)	201 (22.2)	<0.001
Poorer	267 (20.6)	309 (25.7)	230 (25.4)	
Average	332 (25.6)	279 (23.2)	232 (25.6)	
Wealthier	267 (20.6)	192 (16.0)	156 (17.2)	
Wealthiest	143 (11.0)	188 (15.6)	88 (9.7)	
Missing	*0*	*0*	*0*	
Fever[1]				
Yes	649 (75.1)	384 (48.7)	274 (44.4)	<0.001
No	215 (24.9)	404 (51.3)	343 (55.6)	
Missing	*433*	*415*	*290*	
RDT+				
Yes	1145 (88.4)	948 (79.0)	687 (76.1)	<0.001
No	150 (11.6)	252 (21.0)	216 (23.9)	
Missing	*2*	*3*	*4*	
Anemia				
Severe	48 (4.1)	32 (2.7)	17 (1.9)	0.009
Moderate	249 (21.4)	230 (19.4)	167 (18.8)	
Mild	202 (17.4)	242 (20.4)	197 (22.2)	

	Clinic Visits			
	Time from Baseline Visit[2]			
Participant **characteristics at visits** *no. (%)*	**All visits within first 12 months** n=1297	**All visits between 12 to 24 months** n=1203	**All visits after 24 months** n=907	p-value[3]
Not anemic	662 (57.0)	681 (57.5)	506 (57.0)	
Missing	*136*	*18*	*20*	
Seasonality of visit				
Rainy	885 (68.2)	758 (63.0)	411 (45.3)	<0.001
Dry	412 (31.8)	445 (37.0)	496 (54.7)	
Missing	*0*	*0*	*0*	
P. falciparum PCR +				
Yes	829 (64.6)	636 (53.5)	544 (62.0)	<0.001
Mixed-species	*53 (6.4)*	*42 (6.6)*	*41 (7.5)*	*0.694*
Single-species	*775 (93.6)*	*593 (93.4)*	*503 (92.5)*	
No	455 (35.4)	552 (46.5)	334 (38.0)	
Missing	*13*	*15*	*29*	
P. malariae PCR +				
Yes	53 (4.1)	44 (3.7)	38 (4.3)	0.757
Mixed-species	*35 (66.0)*	*25 (56.8)*	*29 (76.3)*	*0.187*
Single-species	*18 (34.0)*	*19 (43.2)*	*9 (23.7)*	
No	1230 (95.9)	1142 (96.3)	842 (95.7)	
Missing	*14*	*17*	*27*	
P. ovale spp. PCR +				
Yes	34 (2.7)	41 (3.5)	20 (2.3)	0.255
Mixed-species	*19 (55.9)*	*21 (51.2)*	*12 (60.0)*	*0.864*
Single-species	*15 (44.1)*	*20 (48.8)*	*8 (40.0)*	
No	1249 (97.3)	1145 (96.5)	860 (97.7)	
Missing	*14*	*17*	*27*	

[1] Fever was measured at time of clinic visit.

[2] Clinic visits could continue past the end of active surveillance till the end of 2017, for a total of 34 months from the first baseline visit. Clinic visits after 24 months occurred after all household surveys had concluded.

[3] Categorical variables were statistically compared using two-sided chi-squared tests or fishers' exact testing in the case of small frequency counts; continuous variables were compared using a two-sided Kruskal-Wallis test of medians to account for non-normality. p-values were not adjusted for multiple comparisons. Missing data were excluded from statistical tests.

Supplemental Table 5. Estimated parasitemias (p/µL) by malaria species

Parasitemia Estimates (p/µL)		n (infect.)	median	IQR	min - max	p-value[1]	n excluded (rehydrated)[2]
		**Survey Population** – *Household Survey Visits*					
**Pm (p/µL)**	Total	175	22.4	8.5-72.2	0.8-358,246		11
	Mixed	129	21.1	8.5-60.1	1.4-358,246	0.259	8
	Mono	46	28.8	9.2-125	0.8-688		3
**Po (p/µL)**	Total	71	5.8	2.0-28.0	0.6-106,476		7
	Mixed	56	5.7	2.3-23.0	0.6-106,476	0.662	5
	Mono	15	9.5	1.7-92.2	0.8-252		2
**Pf (p/µL)**	Total	1760	52.6	8.2-343.3	0.6-268250.0		216
	Mixed	176	120.5	31.9-496.3	0.8-22925.0	<0.001	12
	Mono	1584	46.9	7.4-311.1	0.6-268250.0		204
		**Clinic sub-Population** – *Symptomatic Clinic Visits*					
**Parasitemia Estimates (p/µL)**		n (infect.)	median	IQR	min - max	p-value[1]	n excluded (rehydrated)[2]
**Pm (p/µL)**	Total	132	36.5	4.7-182	0.6-23,288		3
	Mixed	88	31.8	3.6-157	0.6-2,432	0.239	1
	Mono	44	51.5	15.2-204	1.3-23,288		2
**Po (p/µL)**	Total	93	17.7	4.6-65.8	0.2-2,875		2
	Mixed	51	17.8	4.9-68.6	0.3-1,447	0.740	1
	Mono	42	16.7	4.5-65.5	0.2-2,875		1
**Pf (p/µL)**	Total	1970	2644.3	113.2-16931.1	0.6-1,165,100		39
	Mixed	134	508.7	58.8-8387.1	2.1-119,100	0.001	2
	Mono	1834	2897	126.5-18058.3	0.6-1,165,100		37
		**Total Population** – *Survey and Clinic Visits*					
**Parasitemia Estimates (p/µL)**		n (infect.)	median	IQR	min - max	p-value[1]	n excluded (rehydrated)[2]
**Pm (p/µL)**	Total	307	25.7	7.7-119	0.6-358,246		14
	Mixed	217	22.4	6.9-108	0.6-358,246	0.071	9
	Mono	90	36.5	11.8 -187	0.8-23,288		5
**Po (p/µL)**	Total	164	10.2	2.7-47.4	0.2-106,476		9
	Mixed	107	10.8	2.8-36.4	0.3-106.476	0.465	6
	Mono	57	15.8	2.2-65.8	0.2-2,875		3
**Pf (p/µL)**	Total	3730	266.5	18.8-4525.5	0.6-1,165,100		255
	Mixed	310	190	40.7-1427.4	0.8-119,100	0.209	14
	Mono	3418	279.6	17.4-5014.6	0.6-1,165,100		241

[1] p-values were calculated using two-sided Kruskal-Wallis testing of medians to account for non-normality, and were not adjusted for multiple comparisons. Missing data were excluded from statistical tests.

[2] Samples rehydrated due to evaporated DNA were excluded from parasite density analyses.

Supplementary Table 6. PCR primers, probes, and assay conditions

	**Duplex Assay: *P.* malariae-specific, and *P. ovale*-specific, 18S rRNA genes**		
Adapted from:	Shokoples et al. J Clin Microbiology 2009. 47(4): 975-980. (Secondarily from: Rougemont M et al. *J Clin Microbiology 2004*. 42(12):5636-43)		
**Forward Primer – *Pm*** (5'->3')	CCG ACT AGG TGT TGG ATG ATA GAG TAA A		
**Reverse Primer – *Pm*** (5'->3')	AAC CCA AAG ACT TTG ATT TCT CAT AA		
**Forward Primer – *Po*** (5'->3')	CCG ACT AGG TTT TGG ATG AAA GAT TTT T		
**Reverse Primer – *Po*** (5'->3')	AAC CCA AAG ACT TTG ATT TCT CAT AA		
**Probe – *Pm*** (5'->3')	FAM-CTA TCT AAA AGA AAC ACT CAT-MGBNFQ		
**Probe – *Po*** (5'->3')	VIC-CGA AAG GAA TTT TCT TAT T-MGBNFQ		
**Cycling conditions:**	Temp	Duration	No. Cycles
	50C	2 min	x1
	95C	10 min	x1
	95C	15 sec	x45
	60C	1 min	
Reaction conditions:	Roche FastStart Universal Probe Master (Rox)		
	Fwd primers	20µM	
	Rev primers	20µM	
	Probes	20µM	
	DNA	2 µL	
	Total volume	12 µL	
	***P. falciparum*-specific lactate dehydrogenase (*pfldh*) gene**		
Adapted from:	Pickard AL et al. Antimicrob. Agents Chemo 2003. 47(8):2418-2423.		
Forward Primer (5'->3')	ACGATTTGGCTGGAGCAGAT		
Reverse Primer (5'->3')	TCTCTATTCCATTCTTTGTCACTCTTTC		
Probe (5'->3')	FAM/ AGTAATAGTAACAGCTGGATTTACCAAGGCCCCA /TAMRA		
Cycling conditions:	Temp	Duration	No. Cycles
	50C	2 min	x1
	95C	10 min	x1
	95C	15 sec	x40
	60C	1 min	
Reaction conditions:	Roche FastStart Universal Probe Master (Rox)		
	Fwd primer	200nM	
	Rev primer	200nM	
	Probe	100nM	
	Template	2 µl	
	Total volume	12 µl	

[1.] Duplex PCR was carried out to 45 cycle thresholds for *P. malariae* and *P. ovale* spp. detection; however, samples were considered positive if amplification under 40 Cts only, due to observed variability in this assay at later cycle thresholds.

Supplemental Table 7. List of R packages used in analyses.

R package	Version	Citation
tidyverse	v 1.3.2	Wickham H, Averick M, Bryan J, et al. Welcome to the tidyverse. *Journal of Open Source Software*, 2019; 4(43): 1686. doi:10.21105/joss.01686.
dplyr	v 1.1.0	Wickham H, François R, Henry L, Müller K, Vaughan D (2023). dplyr: A Grammar of Data Manipulation, 2023. R package version 1.1.0. Accessed at: https://CRAN.R-project.org/package=dplyr.
readxl	v 1.4.1	Wickham H, Bryan J (2022). readxl: Read Excel Files. R package version 1.4.1. Accessed at: https://CRAN.R-project.org/package=readxl.
tableone	v 0.13.2	Yoshida K, Bartel A (2022). tableone: Create'Table 1' to Describe Baseline Characteristics with or without Propensity Score Weights. R package version 0.13.2. Accessed at: https://CRAN.R-project.org/package=tableone.
devtools	v 2.4.5	Wickham H, Hester J, Chang W, Bryan J (2022). devtools: Tools to Make Developing R Packages Easier. R package version 2.4.5. Accessed at: https://CRAN.R-project.org/package=devtools.
PropCIs	v 0.3-0	Scherer R (2018). PropCIs: Various Confidence Interval Methods for Proportions. R package version 0.3-0. Accessed at: https://CRAN.R-project.org/package=PropCIs.
ggplot2	v 3.4.0	H. Wickham. ggplot2: Elegant Graphics for Data Analysis. Springer-Verlag New York, 2016. Accessed at: https://ggplot2.tidyverse.org.
ggbreak	v 0.1.1	S Xu, M Chen, T Feng, L Zhan, L Zhou, G Yu. Use ggbreak to effectively utilize plotting space to deal with large datasets and outliers. Frontiers in Genetics. 2021,12:774846. doi: 10.3389/fgene.2021.774846
ggthemes	v 4.2.4	Arnold J (2021). ggthemes: Extra Themes, Scales and Geoms for 'ggplot2'. R package version 4.2.4. Accessed at: https://CRAN.R-project.org/package=ggthemes.
ggpubr	v 0.5.0	Kassambara A (2022). ggpubr: 'ggplot2' Based Publication Ready Plots. R package version 0.5.0. Accessed at: https://CRAN.R-project.org/package=ggpubr.
haven	v 2.5.2	Wickham H, Miller E, Smith D (2023). haven: Import and Export 'SPSS', 'Stata' and 'SAS' Files. R package version 2.5.2. Accessed at: https://CRAN.R-project.org/package=haven.
plyr	v 1.8.8	Hadley Wickham. The Split-Apply-Combine Strategy for Data Analysis. Journal of Statistical Software, 2011: 40(1), 1-29. Accessed at: https://www.jstatsoft.org/v40/i01/.
survminer	v 0.4.9	Kassambara A, Kosinski M, Biecek P (2021). survminer: Drawing Survival Curves using 'ggplot2'. R package version 0.4.9. Accessed at: https://CRAN.R-project.org/package=survminer.
purrr	v 1.0.1	Wickham H, Henry L (2023). purrr: Functional Programming Tools. R package version 1.0.1. Accessed at: https://CRAN.R-project.org/package=purrr.
stringr	v 1.5.0	Wickham H (2022). stringr: Simple, Consistent Wrappers for Common String Operations. R package version 1.5.0. Accessed at: https://CRAN.R-project.org/package=stringr.
forcats	v 1.0.0	Wickham H (2023). forcats: Tools for Working with Categorical Variables (Factors). R package version 1.0.0. Accessed at: https://CRAN.R-project.org/package=forcats.
gee	v 4.13-25	Carey VJ (2022). gee: Generalized Estimation Equation Solver. R package version 4.13-25. Accessed at: https://CRAN.R-project.org/package=gee.
geepack	v 1.3.9	Højsgaard, S., Halekoh, U. & Yan J. The R Package geepack for Generalized Estimating Equations Journal of Statistical Software, 2006: 15, 2, pp1—11.
janitor	v 2.2.0	Firke S (2023). janitor: Simple Tools for Examining and Cleaning Dirty Data. R package version 2.2.0. Accessed at: https://CRAN.R-project.org/package=janitor.

lme4	v 1.1-31	Bates D, Maechler M, Bolker B, Walker S. Fitting Linear Mixed-Effects Models Using lme4. Journal of Statistical Software, 2015; 67(1), 1-48. doi:10.18637/jss.v067.i01.
lmerTest	v 3.1-3	Kuznetsova A, Brockhoff PB, Christensen RHB (2017). "lmerTest Package: Tests in Linear Mixed Effects Models." Journal of Statistical Software, 82(13), 1-26. doi:10.18637/jss.v082.i13.
ggsurvfit	v 0.2.1	Sjoberg D, Baillie M, Haesendonckx S, Treis T (2022). ggsurvfit: Flexible Time-to-Event Figures. R package version 0.2.1. Accessed at: https://CRAN.R-project.org/package=ggsurvfit.
gtsummary	v 1.7.0	Sjoberg DD, Whiting K, Curry M, Lavery JA, Larmarange J. Reproducible summary tables with the gtsummary package. The R Journal 2021;13:570–80. Accessed at: https://doi.org/10.32614/RJ-2021-053.
tidycmprsk	v 0.2.0	Sjoberg DD, Fei T (2022). tidycmprsk: Competing Risks Estimation. R package version 0.2.0. Accessed at: https://CRAN.R-project.org/package=tidycmprsk.

Supplemental Table 8: Unadjusted prevalence differences for factors possibly associated with *P. malariae* and *P. ovale* infection prevalence - Survey-based population.

Survey-based Population (N=5,659)								
	***P. malariae* infection**				***P. ovale spp.* infection**			
**Characteristic**	**Yes** n	**No** n	**PD**	**95% CI**	**Yes** n	**No** n	**PD**	**95% CI**
**Age**								
<5 years	34	945	0.017	0.004 – 0.031	23	956	0.016	0.006 – 0.027
5-14 years	106	1766	0.039	0.026 – 0.052	36	1836	0.013	0.005 – 0.020
15+ years	46	2762	*Ref.*		19	2789	*Ref.*	
**Sex**								
Female	83	3034	-0.014	-0.025 – -0.003	43	3074	0.000	-0.007 – 0.007
Male	103	2439	*Ref.*		35	2507	*Ref.*	
**Rurality**								
Rural sites	114	2363	0.045	0.035 – 0.054	47	2430	0.018	0.012 – 0.024
Peri-urban sites	70	1783	0.036	0.027 – 0.046	30	1823	0.015	0.009 – 0.022
Urban sites	2	1327	*Ref.*		1	1328	*Ref.*	
**Malaria RDT testing**								
RDT positive	117	1518	0.049	0.035 – 0.063	53	1582	0.025	0.016 – 0.034
RDT negative	67	3954	*Ref.*		25	3996	*Ref.*	
***P. falciparum* coinfection**								
Yes	135	1841	0.051	0.039 – 0.063	59	1917	0.024	0.016 – 0.032
No	51	3631	*Ref.*		19	3663	*Ref.*	
**Fever in past week**								
Yes	29	890	-0.006	-0.018 – 0.006	19	900	0.007	-0.002 – 0.017
No	155	4577	*Ref.*		59	4673	*Ref.*	
**Bed net used prior night**								
Yes	73	2747	-0.013	-0.023 – -0.003	29	2791	-0.007	-0.014 – -0.001
No	113	2726	*Ref.*		49	2790	*Ref.*	
**Seasonality**								
Dry	84	3020	-0.011	-0.020 – -0.002	45	3059	0.002	-0.003 – 0.008
Rainy	102	2453	*Ref.*		33	2522	*Ref.*	
**Wealth Score**								
Poor/poorer	109	2198	0.015	0.000 – 0.030	42	2265	0.002	-0.007 – 0.011
Wealthier/wealthy	39	2143	-0.015	-0.028 – -0.002	17	2165	-0.008	-0.017 – 0.000
Average	38	1132	*Ref.*		19	1151	*Ref.*	

PD: prevalence difference; Ref = referent category.

Supplemental Table 9: Unadjusted prevalence differences for factors possibly associated with *P. malariae* and *P. ovale* infection prevalence - Clinic-based sub-population.

Clinic-based Sub-population (N=3,349)								
	***P. malariae* infection**				***P. ovale spp.* infection**			
**Characteristic**	**Yes**	**No**	**PD**	**95% CI**	**Yes**	**No**	**PD**	**95% CI**
	**n**	**n**			**n**	**n**		
**Age**								
<5 years	30	791	0.006	-0.011 – 0.024	15	806	-0.001	-0.013 – 0.010
5-14 years	68	1216	0.023	0.007 – 0.038	56	1228	0.024	0.010 – 0.039
15+ years	37	1204	*Ref.*		24	1217	*Ref.*	
**Sex**								
Female	70	1875	-0.010	-0.024 – 0.004	50	1895	-0.006	-0.019 – 0.006
Male	65	1339	*Ref.*		45	1359	*Ref.*	
**Rurality**								
Rural sites	94	1664	0.050	0.038 – 0.061	39	1719	0.010	-0.002 – 0.021
Peri-urban sites	39	1057	0.032	0.019 – 0.045	50	1046	0.034	0.017 – 0.051
Urban sites	2	493	*Ref.*		6	489	*Ref.*	
**Malaria RDT testing**								
RDT positive	125	2609	0.029	0.016 – 0.042	82	2652	0.007	-0.006 – 0.021
RDT negative	10	597	*Ref.*		13	594	*Ref.*	
***P. falciparum* coinfection**								
Yes	88	1919	0.008	-0.005 – 0.021	51	1956	-0.010	-0.023 – 0.003
No	47	1292	*Ref.*		44	1295	*Ref.*	
**Fever at time of visit**								
Yes	60	1243	0.001	-0.016 – 0.019	43	1260	-0.002	-0.018 – 0.014
No	42	907	*Ref.*		33	916	*Ref.*	
**Anemia (Any severity)**								
Yes (Mild/Mod./Severe)	68	1294	0.015	0.001 – 0.029	42	1320	0.003	-0.010 – 0.015
No (Not anemic)	62	1757	*Ref.*		51	1768	*Ref.*	
**Anemia by severity level**								
Moderate/Severe	37	698	0.011	-0.006 – 0.029	23	712	0.002	-0.013 – 0.018
Mild/Not anemic	93	2353	*Ref.*		70	2376	*Ref.*	
**Seasonality**								
Dry	61	1261	0.010	-0.003 – 0.023	45	1277	0.009	-0.003 – 0.021
Rainy	74	1953	*Ref.*		50	1977	*Ref.*	
**Wealth Score**								
Poor/poorer	78	1425	0.018	0.001 – 0.034	43	1460	-0.004	-0.019 – 0.012
Wealthier/wealthy	29	990	-0.006	-0.023 – 0.010	26	993	-0.006	-0.023 – 0.010
Average	28	799	*Ref.*		26	801	*Ref.*	

PD: prevalence difference; Ref = referent category.

Supplemental Figure 1 A-D.

a.

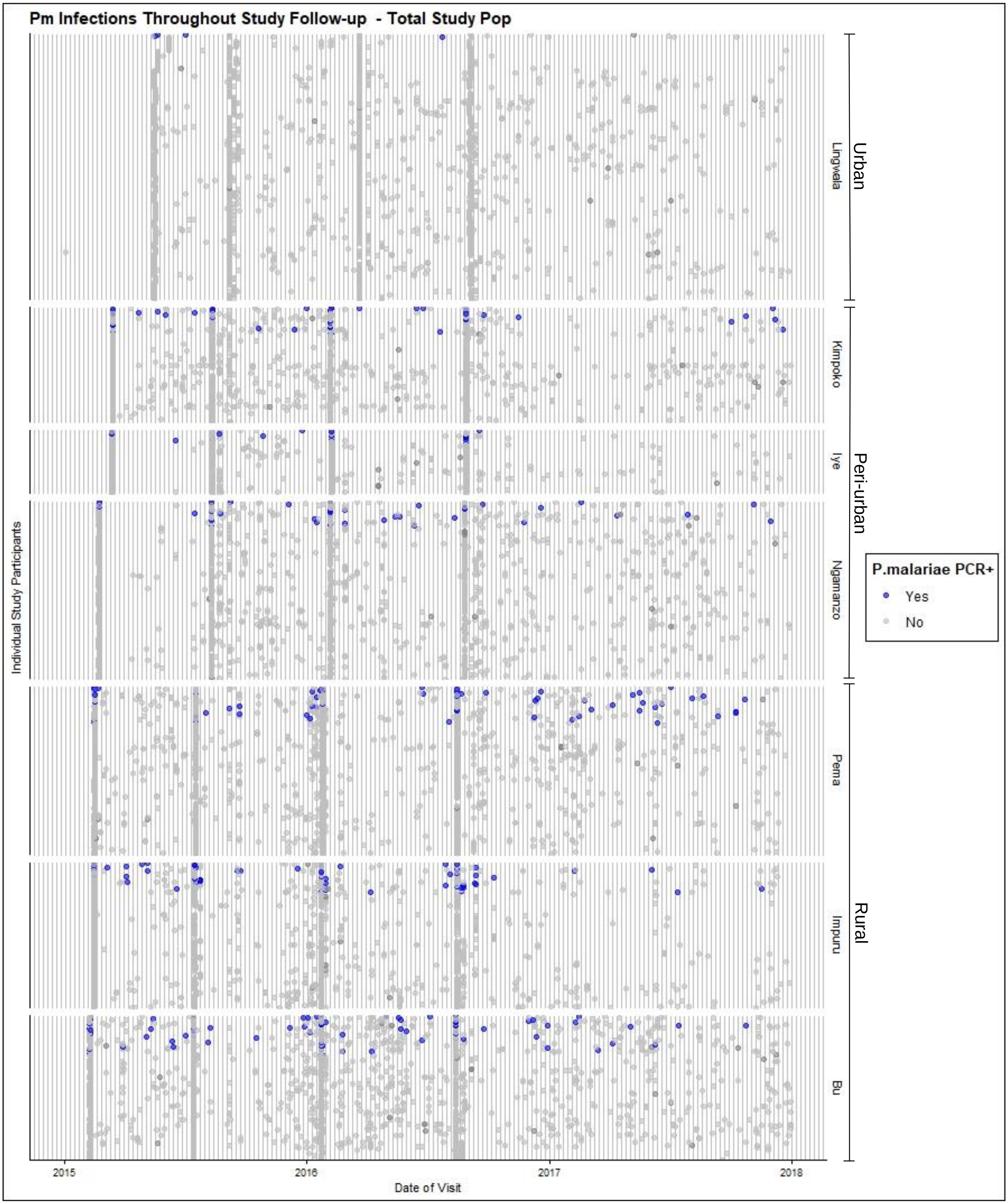

Supplemental Figure 1. *P. malariae* and *P. ovale* spp. infections throughout the study period, within the Total Population, encompassing infections detected at all touch points in the study (survey + clinic visits). a) *P. malariae* infections detected at study visits throughout follow-up, among all subjects in the Total Population. Rows represent individual subjects, sorted by frequency of PCR+ *P. malariae* infections throughout the full study period.

b.

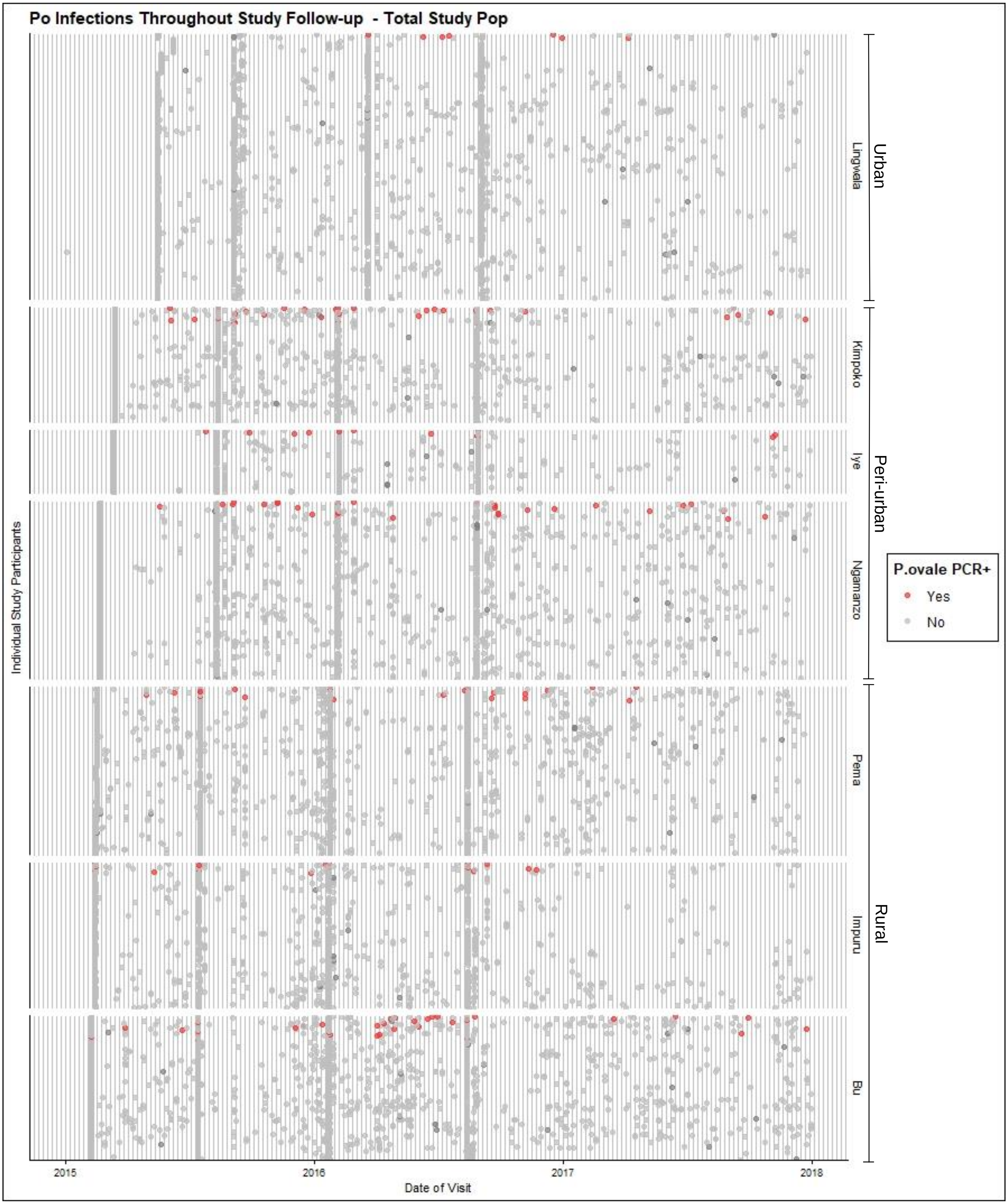

Supplemental Figure 1. ***P. malariae*** **and** ***P. ovale*** **spp. infections throughout the study period, within the Total Population, encompassing infections detected at all touch points in the study (survey + clinic visits).** b) *P. ovale* spp. infections detected at study visits throughout follow-up, among all subjects in the Total Population. Rows represent individual subjects, sorted by frequency of PCR+ *P. ovale* spp. infections throughout the full study period.

c.

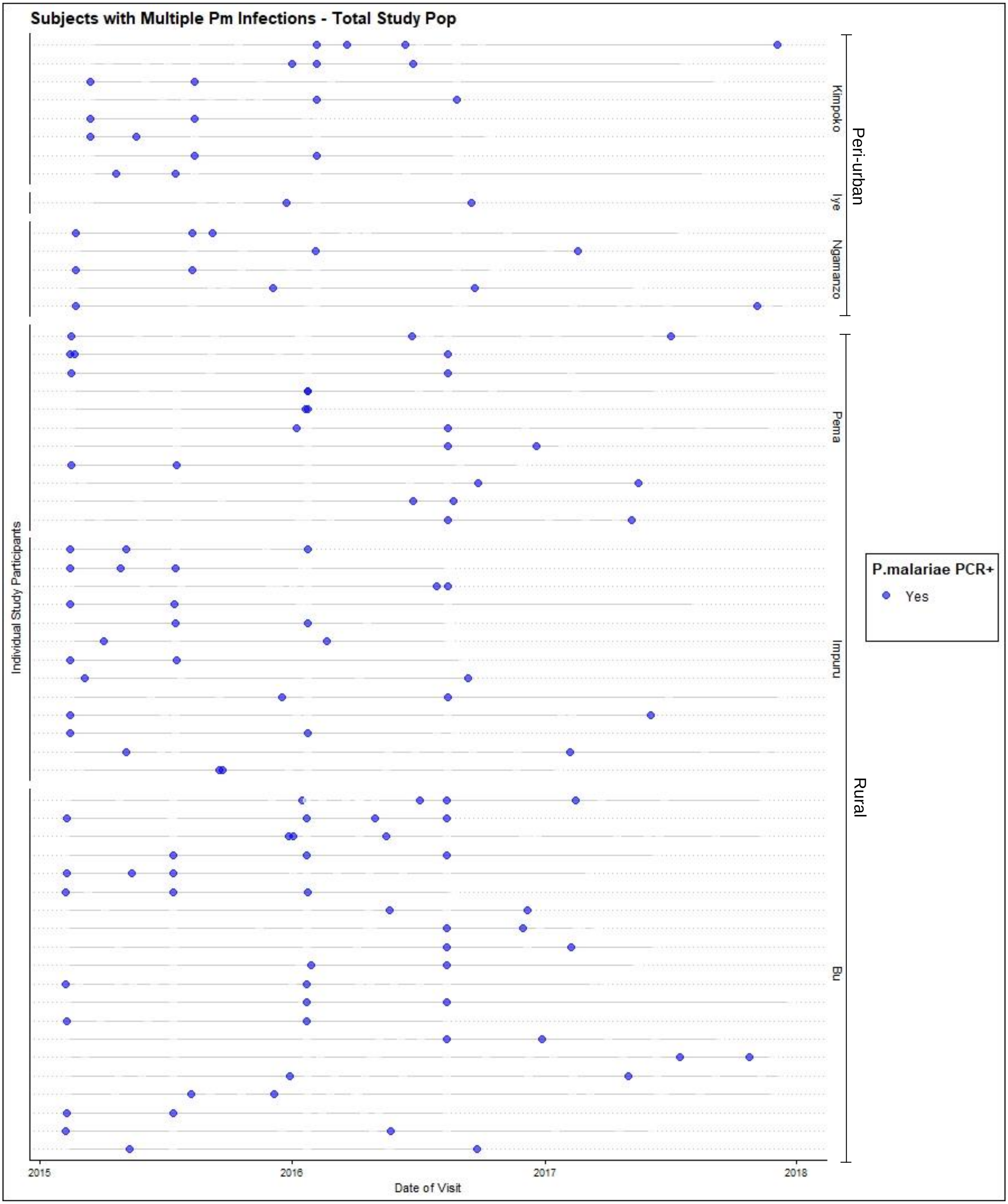

Supplemental Figure 1. *P. malariae* and *P. ovale* spp. infections throughout the study period, within the Total Population, encompassing infections detected at all touch points in the study (survey + clinic visits). c) Multiple *P. malariae* infections detected at study visits throughout follow-up, among all subjects in the Total Population <u>who had at least one *P.* malariae infection during the study</u>. Rows represent individual subjects, sorted by frequency of PCR+ *P. malariae* spp. infections throughout the full study period.

d.

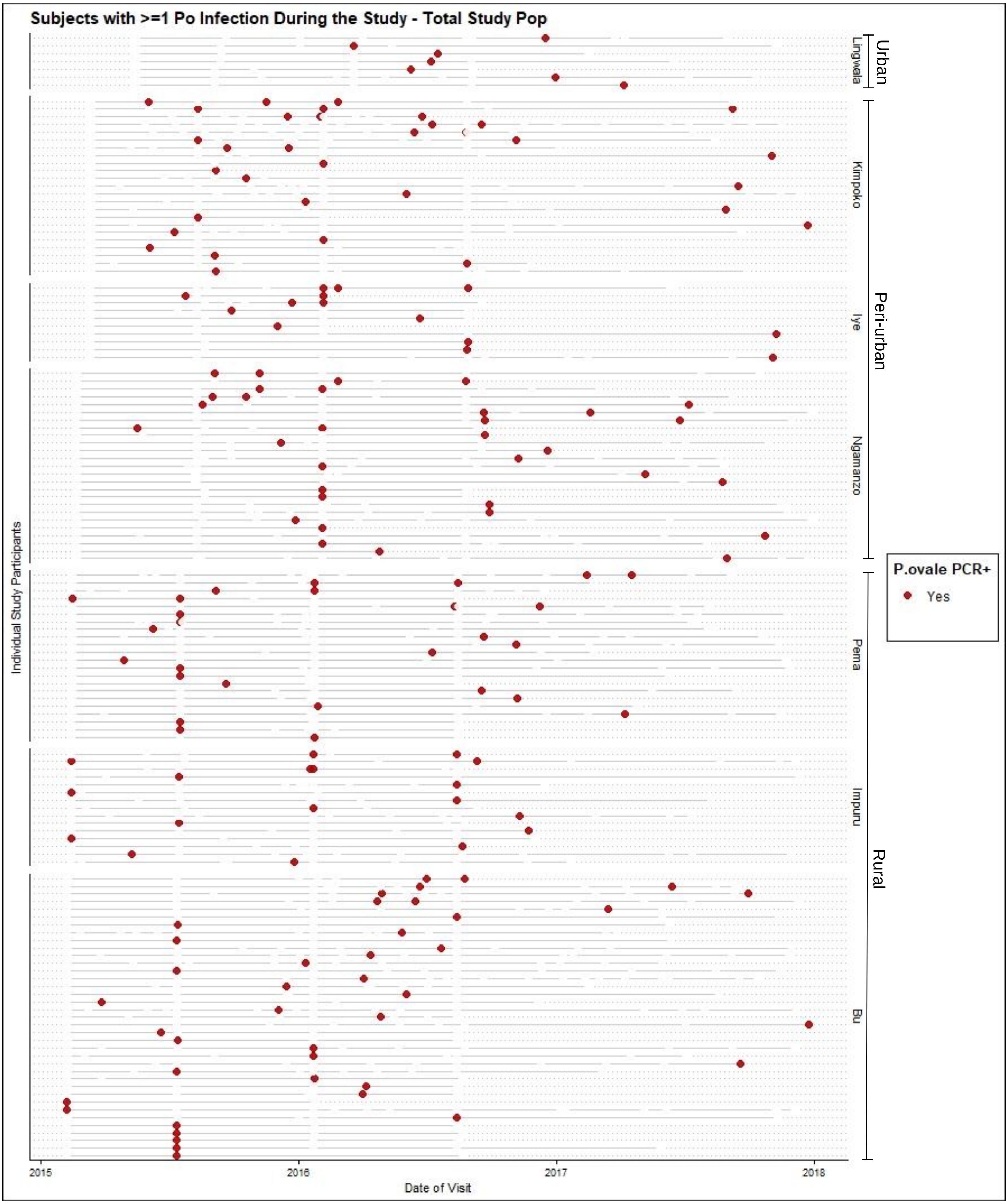

Supplemental Figure 1. *P. malariae* and *P. ovale* spp. infections throughout the study period, within the Total Population, encompassing infections detected at all touch points in the study (survey + clinic visits). d.) Multiple *P. ovale* spp. infections detected at study visits throughout follow-up, among all subjects in the Total Population who had at least one *P. ovale* spp. infection during the study. Rows represent individual subjects, sorted by frequency of PCR+ *P. ovale* spp. infections throughout the full study period.

Supplemental Figure 2 A-B.

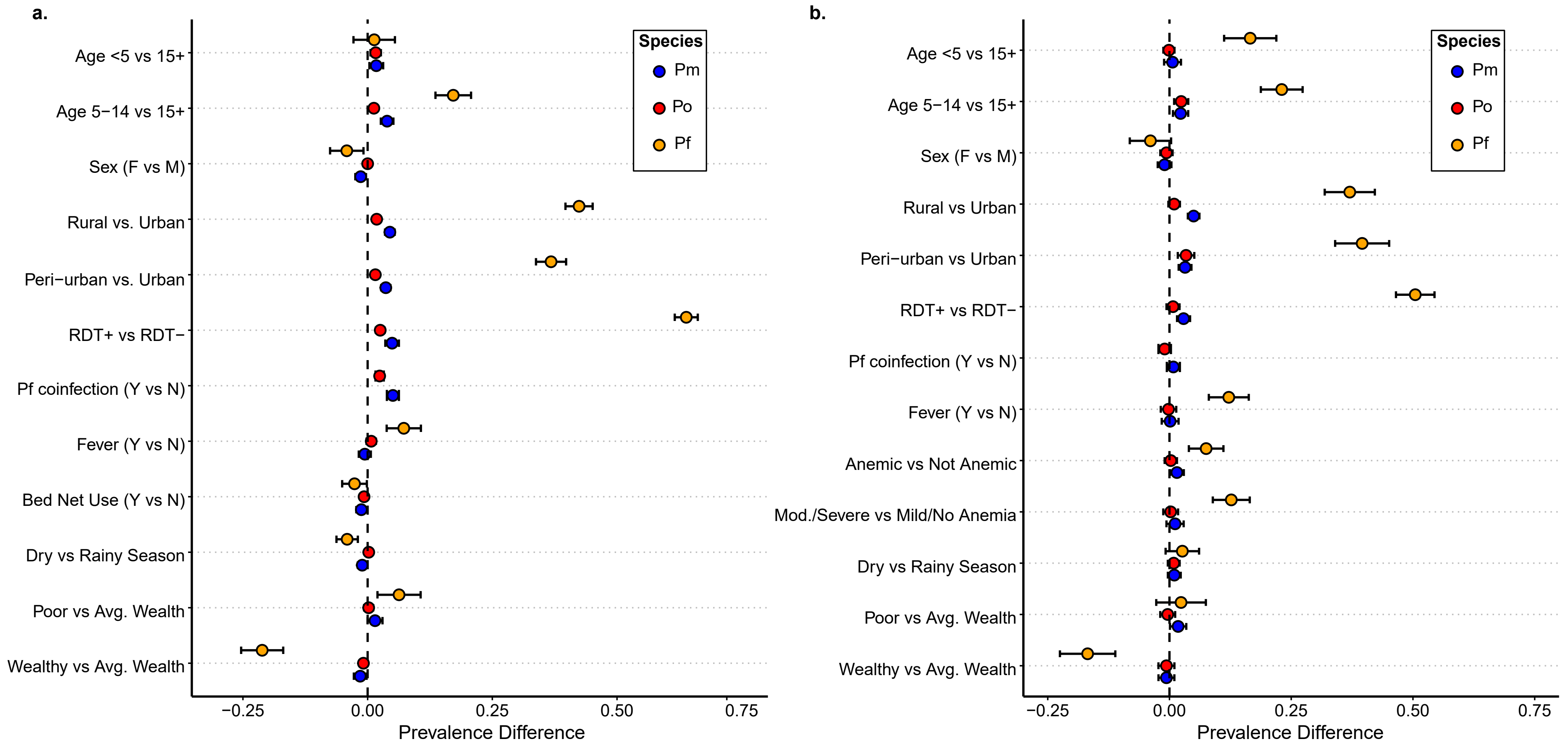

Supplemental Fig. 2. Factors associated with *P. malariae* and *P. ovale* spp. infection prevalence, compared to *P. falciparum*, stratified by study population. a) Factors associated with *P. malariae* (blue) and *P. ovale* (red) infection prevalences compared to *P. falciparum* infection prevalence, at survey visits (baseline and three follow-up surveys). b) Factors associated with *P. malariae* (blue) and *P. ovale* (red) infection prevalences compared to *P. falciparum* infection prevalences, at clinic visits throughout follow-up. Error bars represent 95% CIs around prevalence difference measures. n=5,659 *P. malariae* and *P. ovale* samples and n=5,660 *P. falciparum* samples in the survey population (a), and n=3,349 *P. malariae* and *P. ovale* samples, and n=3,350 *P. falciparum* samples in the clinic-based population (b). CI: confidence interval; Pf: *Plasmodium falciparum*; Pm: *Plasmodium malariae*; Po: *Plasmodium ovale* spp.; RDT: rapid diagnostic test.
