# Supplementary material for: Epidemiology of Plasmodium malariae and Plasmodium ovale spp. in Kinshasa Province, Democratic Republic of Congo
Source: Nat Commun. 2023 Oct 19;14:6618. doi: 10.1038/s41467-023-42190-w (PMC10587068; doi:10.1038/s41467-023-42190-w)
Supplement: Supplementary file 3 — Reporting Summary [file 41467_2023_42190_MOESM3_ESM.pdf]

nature portfolio

Corresponding author(s): Rachel Sendor: rachel.sendor@unc.edu
Jonathan Parr: jonathan_parr@med.unc.edu

Last updated by author(s): Aug 20, 2023

Reporting Summary

Nature Portfolio wishes to improve the reproducibility of the work that we publish. This form provides structure for consistency and transparency in reporting. For further information on Nature Portfolio policies, see our Editorial Policies and the Editorial Policy Checklist.

Statistics

For all statistical analyses, confirm that the following items are present in the figure legend, table legend, main text, or Methods section.

n/a	Confirmed	
☐	☒	The exact sample size (*n*) for each experimental group/condition, given as a discrete number and unit of measurement
☐	☒	A statement on whether measurements were taken from distinct samples or whether the same sample was measured repeatedly
☐	☒	The statistical test(s) used AND whether they are one- or two-sided<br>*Only common tests should be described solely by name; describe more complex techniques in the Methods section.*
☐	☒	A description of all covariates tested
☐	☒	A description of any assumptions or corrections, such as tests of normality and adjustment for multiple comparisons
☐	☒	A full description of the statistical parameters including central tendency (e.g. means) or other basic estimates (e.g. regression coefficient) AND variation (e.g. standard deviation) or associated estimates of uncertainty (e.g. confidence intervals)
☐	☒	For null hypothesis testing, the test statistic (e.g. *F*, *t*, *r*) with confidence intervals, effect sizes, degrees of freedom and *P* value noted<br>*Give P values as exact values whenever suitable.*
☒	☐	For Bayesian analysis, information on the choice of priors and Markov chain Monte Carlo settings
☐	☒	For hierarchical and complex designs, identification of the appropriate level for tests and full reporting of outcomes
☒	☐	Estimates of effect sizes (e.g. Cohen's *d*, Pearson's *r*), indicating how they were calculated

Our web collection on statistics for biologists contains articles on many of the points above.

Software and code

Policy information about availability of computer code

Data collection	Code was not used for data collection in this study.
Data analysis	Dataset construction and cleaning were performed using SAS (v 9.4), and analyses and data visualization were conducted in R (v 4.0.2). Custom R code was generated for analysis and is publicly available on GitHub at: https://github.com/IDEELResearch/nonfalciparum_in_DRC<br><br>The full list of open-source R packages used in this study are as follows: tidyverse (v 1.3.2); dplyr (v 1.1.0) ; readxl (v 1.4.1); tableone (v 0.13.2); devtools (v 2.4.5); PropCIs (v 0.3-0); ggplot2 (v 3.4.0); ggbreak (v 0.1.1); ggthemes (v 4.2.4); ggpubr (v 0.5.0); haven (v 2.5.2); plyr (v 1.8.8); survminer (v 0.4.9); purrr (v 1.0.1); stringr (v 1.5.0); forcats (v 1.0.0); gee (v 4.13-25); geepack (v 1.3.9); ggExtra (v 0.10.0); janitor (v 2.2.0); lme4 (v 1.1-31); lmerTest (v 3.1-3); ggsurvfit (v 0.2.1); gtsummary (v 1.7.0); tidycmprsk (v 0.2.0).

For manuscripts utilizing custom algorithms or software that are central to the research but not yet described in published literature, software must be made available to editors and reviewers. We strongly encourage code deposition in a community repository (e.g. GitHub). See the Nature Portfolio guidelines for submitting code & software for further information.

Data

Policy information about availability of data

All manuscripts must include a data availability statement. This statement should provide the following information, where applicable:

- Accession codes, unique identifiers, or web links for publicly available datasets
- A description of any restrictions on data availability
- For clinical datasets or third party data, please ensure that the statement adheres to our policy

A de-identified version of the study analysis dataset is publicly available through the Carolina Digital Repository at: https://doi.org/10.17615/kjjg-7a88. Dates and additional identifiable variables have been removed from the public dataset for confidentiality of protected health information.

Research involving human participants, their data, or biological material

Policy information about studies with human participants or human data. See also policy information about sex, gender (identity/presentation), and sexual orientation and race, ethnicity and racism.

Reporting on sex and gender	Participant sex was categorized in this study according to available response options on the baseline survey questionnaire of either male or female. Participants self-reported male or female. Main analyses were stratified by male vs. female to report sex-specific differences.
Reporting on race, ethnicity, or other socially relevant groupings	All study participants were recruited from Kinshasa, Democratic Republic of Congo, comprising an entirely African population. Race was not collected in the study survey as all participants were of African descent; participant identification within different local ethnic tribes was collected in the survey. However, we did not analyze differences by ethnic tribe in this specific study.
Population characteristics	The research sample comprises males and females of all ages who live within enrolled households across 7 villages in Kinshasa Province, Democratic Republic of Congo. The study population was comprised of all consenting participants within enrolled households who had a dried blood spot sample available for PCR analysis at the baseline visit (n= l, 565). At the baseline survey, the Survey Population was 55.1% female, and was a median of 14 years of age, with 19.3% of participants aged 0-5 years, 31. 9% aged 5-14 years, and 48.8% aged 15 and older at baseline. Additionally, 42. 4% of the Survey Population lived in households in the 3 rural villages, 33.0% lived in households in the 3 peri-urban villages, and 24.6% lived in households within the urban village in the study. At Baseline, 27% of all participants had a positive malaria rapid diagnostic test, 45% reported sleeping under a bed net the prior night, and approximately 40% of participants were classified as poorest / poorer wealth, 40% as richer/richest wealth, and 20% having average wealth.<br><br>From among the 1, 565 enrolled participants across 242 households, 1,050 {67.1%) of these were also included in the Clinic Subpopulation sample because they visited the study health clinics presumably for experiencing malaria symptoms as instructed during study enrollment, at least once after Baseline through Dec. 31, 2017 outside of the defined household survey follow-ups. All participants were eligible to visit study health clinics; however, some differences were observed between those who ultimately did vs. did not ever visit the study clinic (Supplementary Table 2), including a significantly higher proportion of children 0-5 years and 5-14 years old (p<0.001), a higher proportion of participants in the 3 rural villages and lower proportion of participants in the urban village (p<0.001), and a higher proportion of participants who fell in the poorest household wealth quintile and average wealth quintile, and lower proportion of participants who fell in the wealthiest household wealth quintile (p= 0.011). However, despite differences between those who did vs. did not ever attend a study health clinic for symptoms of malaria in the study, the proportions of subjects across categories were similar between those in the Total Survey population, and those who ever attended a study health clinic visit (Supplemental Table 2). Therefore, we observed that the Clinic Subpopulation appeared generally representative of the broader Total Survey Population.
Recruitment	Enrollment in the study occurred by household. Households were selected through a process in which two GPS points were selected (a beginning point and end point) within each of the 7 villages (within 3 health areas) chosen for inclusion in the study. Recruitment of households was conducted randomly in relation to the GPS points, where 25 households closest to the starting point were selected, moving outwards to the end GPS point. Household eligibility criteria included: accessible by vehicle or short walk from vehicle, non-transient population in the identified site, and a sufficient number of household members who could provide consent to the study. Household members identified for screening were then considered for eligibility criteria, including: permanent residence in the study site, provision of informed consent, and no evidence of impaired judgment for informed consent, ability to understand languages of the study team (local), and no serious illness at the time of screening.<br><br>Three local health facilities were also included in our study, one per health area. The chosen facility was the main government clinic in two of these health areas (Bu – rural, Kimpoko – peri-urban). In urban, Voix de Peuple, one private clinic was selected due to the diverse patient population it served. Only households within the clinic catchment areas that were located within 5km from the clinic were eligible for the study.<br><br>These criteria are outlined in additional detail in Mwandagalirwa et al. Individual and household characteristics of persons with Plasmodium falciparum malaria in sites with varying endemicities in Kinshasa Province, Democratic Republic of the CongoMalar J (2017) 16:456.<br><br>No information was collected during recruitment on reasons for non-participation, or rates of non-participation. It is possible that generalizability of the study could be impacted by self-selection bias if those who elected not to enroll in the study did so for reasons such as being sick at the time of the study visit. This could result in the study population being artificially healthy

compared to the total population we intend to make inferences on with this study. However, as no information was collected on this, we are unable to measure whether this is an issue biasing the study.

Ethics oversight

Informed consent, and assent where required, was obtained from all participants or their legal guardians prior to study enrollment and sampling. The study was approved by the Institutional Review Boards at the University of North Carolina at Chapel Hill (IRB#: 14-0489) the University of Iowa (#201701201), and the Kinshasa School of Public Health (ESP/CE/015/014).

Note that full information on the approval of the study protocol must also be provided in the manuscript.

Field-specific reporting

Please select the one below that is the best fit for your research. If you are not sure, read the appropriate sections before making your selection.

☐ Life sciences ☒ Behavioural & social sciences ☐ Ecological, evolutionary & environmental sciences

For a reference copy of the document with all sections, see nature.com/documents/nr-reporting-summary-flat.pdf

Behavioural & social sciences study design

All studies must disclose on these points even when the disclosure is negative.

Study description

This study was a longitudinal cohort study assessing the epidemiology of malaria infection among males and females of all ages who live in enrolled households within 7 villages across Kinshasa Province, Democratic Republic of Congo. Household surveys were performed at baseline (starting in Feb. 2015), and at each of 3 biannual follow-up time points. After baseline, participants were instructed to visit study health clinics if they experienced malaria symptoms at any point during the study follow-up period; clinic visits were allowed to continue past the final household follow-up visit, through to the end of 2017. Dried blood spot samples were collected from all participants who consented, at all study touch points (i.e., all household surveys, and each time a participant visited study health clinics, if applicable). Dried blood spots were stored with dessicant and shipped to the University of North Carolina for DNA extraction and polymerase chain reaction (PCR) to identify Plasmodium falciparum malaria infection (reported qualitatively as P. falciparum infection [Yes vs. No], and quantitatively as parasitemia amount), and non-falciparum malaria infections for P. malariae and P. ovale species (reported qualitatively as P. malariae and P. ovale spp. infection [Yes vs. No] and semi-quantitatively as the approximate parasitemia amount per sample; these are semi-quantitiative as the 18S rRNA gene target is a multi-copy target that can vary by parasite). Non-falciparum parasitemias were estimated using comparison of clinical samples against standard dilution curves of positive control samples with known parasitemia concentrations.

We estimated the incidence and prevalence of plasmodium species in this population, overall, and among participant factors of interest.

Research sample

The research sample comprises males and females of all ages who live within enrolled households across 7 villages in Kinshasa Province, Democratic Republic of Congo. This dataset and the associated clinical samples were collected previously, between 2015-2017, and these existing datasets were used for the present study analysis where they were linked to new molecular analyses (PCR) of the existing, previously-extracted DNA that had been retained for future research (with the DNA samples stored at -80 Celsius at the University of North Carolina).

The study sample was comprised of all consenting participants within enrolled households who had a dried blood spot sample available for PCR analysis at the baseline visit (n=1,565). These participants were then followed longitudinally for an additional 3 follow-up household surveys (biannually), with some loss-to-follow-up observed. These participants comprised the "Survey Population" sample. At the baseline survey, the Survey Population was 55.1% female, and was a median of 14 years of age, with 19.3% of participants aged 0-5 years, 31.9% aged 5-14 years, and 48.8% aged 15 and older at baseline. Additionally, 42.4% of the Survey Population lived in households in the 3 rural villages, 33.0% lived in households in the 3 peri-urban villages, and 24.6% lived in households within the urban village in the study.

From among the 1,565 enrolled participants across 242 households, 1,050 (67.1%) of these were also included in the Clinic Subpopulation sample because they visited the study health clinics presumably for experiencing malaria symptoms as instructed during study enrollment, at least once after Baseline through Dec. 31, 2017 outside of the defined household survey follow-ups. All participants were eligible to visit study health clinics; however, some differences were observed between those who ultimately did vs. did not ever visit the study clinic (Supplementary Table 2), including a significantly higher proportion of children 0-5 years and 5-14 years old (p<0.001), a higher proportion of participants in the 3 rural villages and lower proportion of participants in the urban village (p<0.001), and a higher proportion of participants who fell in the poorest household wealth quintile and average wealth quintile, and lower proportion of participants who fell in the wealthiest household wealth quintile (p=0.011). However, despite differences between those who did vs. did not ever attend a study health clinic for symptoms of malaria in the study, the proportions of subjects across categories were similar between those in the Total Survey population, and those who ever attended a study health clinic visit (Supplemental Table 2). Therefore, we observed that the Clinic Subpopulation appeared generally representative of the broader Total Survey Population.

The Total Survey Population was assumed to be fairly representative of the overall study population, within each of the villages, as households were enrolled through an approximately randomized manner within each of the 7 villages. Randomization of households for enrollment is reported in additional detail under the "Recruitment" field below.

The study sample was chosen because of the high prevalence of malaria infection in the Democratic Republic of Congo. 7 sites were selected for enrollment in order to increase diversity of populations across environment, elevation, urbanicity, malaria endemicity

and transmission risk, and additional environmental and social factors related to place. From the enrolled sample, only those who had baseline dried blood spots for PCR analysis were included in the study analysis populations as the outcome of interest (malaria infection) was measured by PCR. Participants who had samples at subsequent household surveys but did not have a baseline survey were excluded as incidence estimates could not be confirmed without confirmation that participants were not infected at baseline.

Sampling strategy

Sampling of study sites was conducted in a semi-randomized manner, as outlined above under 'Recruitment'. Villages were identified within 3 randomly selected Health Areas, where Health Areas were chosen across a spectrum of malaria endemicities. Within villages, a random sample of households was selected through random GPS point identification and subsequent sampling of households that were located nearest to the identified point. Per the protocol, 300 households were targeted for enrollment in order to reach a population sample of approximately 1200 participants in the study. Sample size calculations were performed for the initial cohort study population prior to sampling; however, no sample size calculations were carried out specific to this analysis of the existing cohort study data. For this analysis, we used all available participant data and clinical samples from the cohort study. 95% confidence intervals are reported for estimates in our analysis; no a priori power analyses were conducted for this analysis.

Data collection

Study questionnaires, malaria rapid diagnostic tests, and clinic samples were collected at all household visits and as-needed visits to study health centers. A trained, local field team including clinical personnel performed data collection in the local language, and recorded responses on paper questionnaires. At household survey visits, the local field team, including an interviewer and lab nurse, as well as family members and the participant were in attendance at interviews; at clinic visits, the study nurse, participant, and any family members in attendance at the visit were present for the interview. Responses on paper forms were subsequently entered into a study database. Dried blood spots were collected at visits and shipped to the University of North Carolina for malaria testing. DNA was extracted from punched dried blood spots and underwent PCR testing for P. falciparum, P. malariae, and P. ovale malaria infection. P. falciparum PCR was performed testing 2 samples per subject to confirm infection; P. malariae and P. ovale spp. PCR were performed several years after P. falciparum using the same extracted DNA samples (stored at -80Celcius), and were tested as single samples per subject using a duplex PCR assay. The study field team conducting data entry and sample collection during household and clinic visits were blinded to malaria outcome data as PCR outcome results were generated after the fact using samples collected at the time of the visit. The researcher performing data analyses also conducted the non-falciparum PCR assays, and was blinded to participant data when determining thresholds for PCR results and when linking PCR outcomes to study participant metadata (via a sample ID).

Timing

Study data and clinical samples were collected from participants between February 2015 (start of baseline surveys) and December 2017. Dried blood spot samples were punched and underwent DNA extraction for PCR analysis of P. falciparum malaria infection in 2017. Extracted DNA samples were stored at -80 celcius from 2017 onwards for future research use; these existing DNA samples for all study participants were then used again in 2021 for PCR analysis of P. malariae and P. ovale malaria infections. DNA samples that had evaporated over time were rehydrated (these were excluded from parasitemia analyses in the study due to inability to confirm parasitemia in original sample concentrations). DNA degradation over time is possible, which is expected to impact only the PCR analysis for P. malariae and P. ovale species but not P. falciparum species results. However, the re-analysis of extracted DNA in 2021 was the first time these existing DNA samples were re-used after their initial extraction in 2017, minimizing DNA degredation from multiple freeze-thaw cycles prior to this study.

Data exclusions

We excluded from our study any participant who was enrolled but who did not have a dried blood spot sample available for PCR testing at baseline (n=26 subjects). Data was cut at the end of 2017, excluding a few select study health clinic visits that occurred intermittently in 2018 (n=32 visits, of which 16 were P. falciparum positive). 201 paper records from unscheduled study health clinic visits were unaccounted for during the study and were not included; these are assumed to be missing at random as they were not associated with a specific clinic or village. Participants were also excluded if their PCR results could not be linked to cohort metadata due to typos or errors in subject IDs.

Non-participation

Among 1,565 subjects included in the study at baseline, 93% were retained through Follow up 1, 84% were retained through Follow 76% were retained through Follow up 3 (final household survey visit). Reasons for loss-to-follow-up were not able to be measured.

67% of subjects in the total survey population visited study health clinics at least once during the study. We are unable to distinguish whether those who never visited clinics did not visit because they did not experience malaria symptoms warranting clinical attention at any point during follow-up, or alternatively if they did not visit due to non-participation in this aspect of the study or they attended non-study affiliated clinics instead.

Randomization

Participants were not randomized in this study. As this was a descriptive study and only crude associations were evaluated, we do not claim any associations as causal. Crude associations only were assessed between participant characteristics and prevalence of malaria species infections. We did not adjust for covariates for this descriptive study.

Reporting for specific materials, systems and methods

We require information from authors about some types of materials, experimental systems and methods used in many studies. Here, indicate whether each material, system or method listed is relevant to your study. If you are not sure if a list item applies to your research, read the appropriate section before selecting a response.

Materials & experimental systems

n/a	Involved in the study
☒	☐ Antibodies
☒	☐ Eukaryotic cell lines
☒	☐ Palaeontology and archaeology
☒	☐ Animals and other organisms
☒	☐ Clinical data
☒	☐ Dual use research of concern
☒	☐ Plants

Methods

n/a	Involved in the study
☒	☐ ChIP-seq
☒	☐ Flow cytometry
☒	☐ MRI-based neuroimaging
